# Interoceptive sensitivity, body weight and eating behavior in children: a prospective study

**DOI:** 10.3389/fpsyg.2014.01003

**Published:** 2014-09-09

**Authors:** Anne Koch, Olga Pollatos

**Affiliations:** ^1^Department of Psychology, Faculty of Human Sciences, University of PotsdamPotsdam, Germany; ^2^Department of Health Psychology, Institute of Psychology and Education, Ulm UniversityUlm, Germany

**Keywords:** body weight, children, eating behavior, heartbeat perception, interoceptive sensitivity, overweight

## Abstract

Previous research indicates that interindividual differences in the ability to perceive one's own bodily signals (interoceptive sensitivity, IS) are associated with disordered eating behavior and weight problems. But representative and prospective data in children are lacking and therefore, the exact nature of these observed associations remains unclear. Data on IS measured by heartbeat perception ability in 1657 children between 6 and 11 years of age were collected on the basis of two measurement points with a year distance in time. Stability of the construct and its prospective association with different food approach behaviors [assessed via parent questionnaires (Children's Eating Behavior Questionnaire and Dutch Eating Behavior Questionnaire)] as well as with weight status were analyzed via structural equation modeling. Main results were that only in overweight children external and emotional eating behavior were predictive for later IS, whereas no such relation was found in normal weight children. There was no direct relation between IS and body mass index. For the first time, we could show that eating behavior and IS in middle childhood are prospectively related to each other. But surprisingly, our data indicate that altered interoceptive processes rather follow than precede non-adaptive eating behavior patterns in overweight children. This suggests a possible crucial role of faulty learning mechanisms in eating behavior early in life, undermining the later confidence in one's body.

## Introduction

Examining the factors that influence children's overweight and eating behavior is of great relevance given the rising prevalence of overweight and obesity in childhood. One possible associated intrapersonal factor is a low ability to perceive and process own inner bodily signals or states, also known as *interoceptive sensitivity* (IS; Craig, 2002; Herbert et al., 2013; Herbert and Pollatos, 2014). Interoception includes the perception of physical sensations related to internal organ function, such as respiration, heartbeat or satiety (Vaitl, 1996), it has been linked with activations in specific brain areas including the right insula (Craig, 2009) and its essential role for emotion processing, emotion regulation as well as decision-making has repeatedly been demonstrated (Bechara and Naqvi, 2004; Pollatos et al., 2005; Füstös et al., 2013; Terasawa et al., 2013). But being *interoceptively sensitive* to body signals on or beyond a conscious level is not automatically identical to being *interoceptively aware*, since not all interoceptive information enters consciousness and whether we become subjective aware of them, evaluate them and whether we act according to them depends strongly on cognitive processes including attention, appraisal, beliefs, memories, or attitudes (Mehling et al., 2009). Thus, an accurate perception of bodily sensations and being aware/confident of these bodily changes in terms of how to interpret and/or handle them seem to represent distinct processes and should not be conflated.

However, most of the research so far has investigated interoceptive processes in relation to eating behavior and weight by referring to the term *interoceptive awareness* (Fassino et al., 2004; Merwin et al., 2010). In her psychosomatic theory, Bruch was the first who suggested that patients with eating disorders and/or obesity “have in common the inability to identify hunger correctly or to distinguish it from other states of bodily need or emotional arousal” (Bruch, 1973, p. 45). This illustrates the common use of interoceptive awareness (IA) as a metacognitive construct, addressing difficulties in the *identification* or *interpretation* of internal [emotional and gastrointestinal (such as hunger and satiety)] stimuli (Jacobi et al., 2004). Again, it is important to note that there is a distinct and clear difference between IS and IA, though both expressions were used from researchers of both areas in almost the identical way (see Garfinkel and Critchley, 2013 for further information).

There is little question that eating disorder patients and overweight persons suffer from low IA as assessed by questionnaires and demonstrated in numerous cross-sectional studies in different age groups (e.g., Leon et al., 1993; Golay et al., 1997; Fassino et al., 2004; Sim and Zeman, 2004; Matsumoto et al., 2006; Clausen et al., 2011). Most studies used the subscale *interoceptive awareness* of the Eating Disorder Inventory (EDI), a questionnaire that has become a standard tool in investigating behavioral and psychological characteristics of eating disorders in children, adolescents and adults (Garner et al., 1983; Garner, 1991, 2004). However, longitudinal evidence on the role of interoceptive processes for eating behavior is scarcer. Low IA was found to be predictive of disordered eating in adolescent girls (Leon et al., 1995), and a similar result was obtained by Gustafsson et al. (2010) who reported that higher IA seemed to be a protective factor against the development of disordered eating in adolescent girls. Likewise, deficits in IA were found to be predictive of illness severity 5–10 years later in adult anorexic patients (Bizeul et al., 2001). In other studies that considered a multivariate model, IA was not predictive for eating disorder symptoms in the total sample of adolescents (Killen et al., 1994, 1996; Leon et al., 1999) but univariate differences were found by comparing eating disorder symptomatic and asymptomatic groups (Killen et al., 1994, 1996). Therefore, the role of IA as risk factor is still described as unclear and variable in its specificity. One possible reason for that might be that the IA EDI subscale does not assess one single construct. It comprises two interrelated but rather distinct dimensions: deficits in awareness and identification of emotions and deficits in awareness and identification of hunger and satiety. This dichotomy is supported by a relatively weak internal consistency of the IA scale (Eberenz and Gleaves, 1994). Further, this scale might be limited in its assessment of true IA and in its reliance on self-report, since individuals with deficits in awareness may not be able to accurately report on these deficits and vice versa.

Besides considering IA via self-report questionnaires, few studies have begun to investigate the rather “pure” physiological aspect of interoception, that is the sensitivity to bodily signals, IS, in relation to eating behavior and weight in adults. The study of Herbert and Pollatos (2014) lately showed reduced IS in overweight and obese individuals as well as an inverse correlation between BMI and IS in this group, while no such relationship was observed for normal weight individuals. Concerning eating pathologies, Pollatos et al. (2008) found decreased IS in female anorexia nervosa patients, which was not correlated with the IA EDI subscale, whereas Klabunde et al. (2013) found deficits in IS in female patients recovered from bulimia nervosa. In contrast to this, Eshkevari et al. (2014) recently reported no IS deficits, but greater IA problems in females with eating disorders. Lately, Herbert et al. (2013) showed that higher IS significantly predicted higher *intuitive eating* and especially those eating facets that have been suggested to be associated with the awareness of hunger and satiety and the willingness to eat in order to satisfy hunger rather than to eat for external and emotional reasons. Furthermore, Herbert et al. (2013) found that IS negatively predicted body mass index (BMI). Moreover, the study of Ainley and Tsakiris (2013) demonstrated that IS was significantly negatively correlated with self-objectification; the tendency to regard one's body and self primarily as “objects” from the outside, valuing appearance over function, a characteristic typically found in eating disorders.

All of these studies used standard tasks to assess IS deficits, heartbeat perception tasks that measure the correspondence between actual heart rate and subjective judgment (Vaitl, 1996). There are two main approaches for the task: the *signal detection or tracking method*, originally proposed by Schandry (1981; Dunn et al., 2007; Herbert et al., 2007; Pollatos et al., 2009; Ainley et al., 2012), which assesses one's accuracy in detecting his or her heartbeats by counting them in a given time interval and the *signal discrimination method*, which presents a series of external stimuli (typically tones or lights) and requires the participant to judge whether the stimuli are simultaneous with his or her own heartbeat (Whitehead and Drescher, 1980; Eshkevari et al., 2014). While the detection task is widely used, it has been suggested to be influenced by expectancies or guesses about heart rate or other factors such as attention or motivation (e.g., Windmann et al., 1999). Nevertheless, a convincing body of evidence exists showing that both methods correlate with each other and are congruent with effects of IS on emotions (for reviews see Vaitl, 1996; Wiens, 2005). It could be demonstrated that heartbeat perception is associated with a more finely tuned self-regulation of behavior according to one's bodily needs (Herbert et al., 2007) and that it correlates with the ability to detect changes in other autonomically innervated organs, such as the activity of the stomach (Whitehead and Drescher, 1980; Herbert et al., 2012b). This highlights its role as an indicator of a generalized sensitivity for visceral processes in situations evoking interoceptive signals (Herbert and Pollatos, 2012), even during food deprivation and feeling hungry (Herbert et al., 2012a). Recently, for the first time, we presented a modified child version of the tracking method assessing heartbeat perception in relation to physical and emotional parameters in a large group of children (Koch and Pollatos, 2014). However, longitudinal data on heartbeat perception is completely missing so far in any age group, although this would be very important to consider in terms of interrelations between IS, body weight and eating behavior.

To summarize, the perception and processing of internal body signals seems to be a crucial factor for eating behavior and body weight, most of the research has investigated this relation via self-reported IA, only a few cross-sectional studies used a more objective physiological measure of IS via heartbeat perception, merely in adult females. Although in all of these studies IS has been hypothesized or described as a possible biological trait or preceding factor for eating behavior and body weight (Pollatos et al., 2008; Ainley and Tsakiris, 2013; Herbert et al., 2013; Klabunde et al., 2013; Herbert and Pollatos, 2014), nothing is known about its relevance and its development in children. Furthermore, questions remain as to whether interoceptive processes are cause or consequence, and whether they should be regarded as biological, perceptual, or cognitive problem, in terms of whether they are innate or modifiable or whether they relate primarily to pure detection or interpretation deficits or both.

Research on eating behavior in children and especially the one on the etiology of childhood overweight or obesity usually refers to the already mentioned psychosomatic theory (Bruch, 1964) as well as the externality theory (Schachter, 1968, 1971; Rodin, 1981), distinguishing two overeating styles, *emotional overeating* (overeating in response to negative emotions such as anxiety or sadness) and *external overeating* (eating in response to external food cues such as sight and smell, regardless of the internal states of hunger and satiety). Although often co-occurring, both eating behavior styles refer to independent constructs and both might be manifested independently of each other (Van Strien et al., 2009). Emotional overeating with its difficulties in distinguishing hunger from other aversive internal states is possibly a result of inappropriate learning experiences early in life, like parental food controlling practices (e.g., pressure to eat, using food for comfort or for rewarding purposes) in which there is insufficient regard for the real needs of the child (Snoek et al., 2007; Van Strien and Bazelier, 2007; Kröller et al., 2013). Externality theory on the other side focuses on the external (food) environment as a determinant of eating behavior, explaining overeating as a result of an elevated responsiveness to environmental food cues, meanwhile ignoring internal, physiological hunger and satiety signals (Schachter, 1971). For example Jansen et al. (2003) found reduced appetitive responses in normal weight children, whereas overweight children did not adjust their food intake after smelling or tasting food cues.

The Dutch Eating Behavior Questionnaire (DEBQ) is one of the most widely used and validated instruments for assessing eating behavior in children and adults. It explores both emotional as well as external eating and significantly differentiates between obese and non-obese children/adults on these eating styles (Braet and Van Strien, 1997; Franzen and Florin, 1997; Caccialanza et al., 2004; Van Strien and Oosterveld, 2008). Van Strien (2000) found that IA as assessed by questionnaire predicted emotional eating in contrast to external eating in young females. Furthermore, the Children's Eating Behavior Questionnaire (CEBQ; Wardle et al., 2001) is another validated parent-rated questionnaire that assesses a broader spectrum of eating style dimensions, not necessarily independent of one another in children. Four of its eight scales indicate “food approach” behavior and thus positive inclinations for eating, whereby overweight children generally score higher on all of them (Sleddens et al., 2008; Viana et al., 2008; Webber et al., 2009; Santos et al., 2011). Besides the subscale emotional overeating, the scales enjoyment of food and food responsiveness measure an elevated interest in food as well as responsiveness to food cues and the scale desire to drink reflects the desire of children to have drinks to carry around them (Wardle et al., 2001).

The aim of the present study was to shed light on possible relations between IS, body weight and different eating behaviors in children using a longitudinal perspective. Next to the fact that to our knowledge no study investigated these associations so far, we wanted to examine possible causality or directionality between eating behavior and IS using a large representative sample. We examined the interrelationships between individual IS as assessed by a heartbeat perception task and BMI, using a two-wave research design. First, in an exploratory cross-lagged analysis we inspected whether IS had an influence on BMI or vice versa. The second relevant focus of this study centers on the prospective association between IS and children's “food approach” behavior. Given the found results of an elevated “food approach” behavior primarily in overweight, we examined whether the relations between these variables differed relative to BMI-status in a multi-group model. We hypothesized a negative relation between “food approach” behavior styles and IS, especially in overweight children in contrast to normal weight children, whereas the direction of this association was of exploratory nature.

## Materials and methods

### Study design, participants and procedure

Data for this study were retrieved from a longitudinal study on intrapersonal developmental risk factors in childhood and adolescence (*PIER study*), for which approval was obtained from the local Ethics Committee as well as from the Ministry of Education. It was conducted among elementary school children from first to fourth grade in the surrounding area of Potsdam in the federal state of Brandenburg, Germany, after legal guardians had provided written informed consent. The study included two assessments, approximately separated by a 1-year time interval (*M* = 273 days, *SD* = 55 days) and started in 2012 (baseline, Time 1 (T1), see also Koch and Pollatos, 2014). At baseline, 1657 children between 6 and 11 years of age from first to third grade were recruited from 32 elementary schools. At Time 2 (T2) 47 children (2.8%) were absent. Dropout analyses revealed no effects for sex, BMI, educational attainment of the mother or eating behavior (*ps* > 0.10). However, children who had dropped out were significantly younger (*M* = 8.08, *SD* = 0.88) and had a lower heartbeat perception (HBP) score (*M* = 0.46, *SD* = 0.23) compared to those who completed both assessments (*M_age_* = 8.39, *SD_age_* = 0.95; *t*_(1653)_ = 2.19, *p* < 0.05, *d* = 0.34; *M_HBP−Score_* = 0.55, *SD_HBP−Score_* = 0.26; *t*_(1449)_ = 2.00, *p*< 0.05, *d* = 0.33).

At each time point of data collection, children were tested individually with regard to the same various psychological variables in a separate room in school on 2 days within 1 week for 1 h per day, while primary caregivers completed a questionnaire at home. Children received a small gift and a cinema voucher for their participation both times.

In total, participants at T1 were 52.1% female and 47.9% male with a mean age of *M* = 8.38 years (*SD* = 0.95) and at T2 51.9% were female and 48.1% were male with a mean age of *M* = 9.13 years (*SD* = 0.93).

### Heartbeat perception task

As more precisely described in Koch and Pollatos (2014) the heartbeat perception task was performed following the Mental Tracking Method proposed by Schandry (1981) in a modified child version. A short training interval of about 10 s was followed by three intervals of 15, 20, and 18 s, separated by two standard resting periods of 20 s. During each interval, children counted their own heartbeats by concentrating on their heart activity, while they were seated and not permitted to attempt any physical manipulation.

Meanwhile, children's actual cardiac activity was recorded using the mobile heart frequency monitor RS800CX (Polar Electro Oy, Kempele, Finland), a mobile device that enables the easy, non-invasive and –reactive recording of inter-beat-intervals and whose validity and reliability compared to alternative ECG measurement devices could be shown in children and adults (Radespiel-Tröger et al., 2003; Kingsley et al., 2005; Gamelin et al., 2008; Nunan et al., 2008). The strap with the electrodes was attached to both hands and fixed to a table. Signals were sampled at 1000 Hz and analyzed by the corresponding Polar ProTrainer 5 software (version 5.40.172), which relies on the HRV analysis software of the University of Kuopio, Finland (Niskanen et al., 2004).

IS was then determined via the mean score across the three intervals (HBP-Score), calculated according to the following transformation:

1/3 Σ [1 - (|recorded heartbeats - counted heartbeats| /recorded heartbeats)].

Higher scores indicate higher sensitivity to heartbeats, so that the maximum score of 1 indicates absolute accuracy of heartbeat perception and the minimum score of 0 indicates that the child did not perceive any of his or her heartbeats, while a score of 0.5 indicates that on average the child detected every other heartbeat. The internal consistency of the task was excellent both times (Cronbach's α T1: 0.91, T2: 0.90).

### Demographic measure

Mother's self-reported educational attainment was distinguished from 1 (= no educational degree) to 6 (= university degree).

### Body mass index and weight status

Height and weight of each child were determined using standard procedures in light clothing without shoes to the nearest 0.1 kg and 0.1 cm by means of calibrated digital scales and calibrated ultrasound measurement devices. BMI was calculated as the standard ratio of weight in kg divided by the square of height in meters. To correct for age and sex, individual BMI-values were converted to *z*-scores (*BMI-SDS* values, standard deviation score values) based on the national reference data for German children (Kromeyer-Hauschild et al., 2001). Those were used to determine the child's weight status.

In the normal weight group, we incorporated those children who were between 10 and 90th BMI percentile at both time points (*n* = 1215, 629 girls, 586 boys) and in the overweight group those children who were over the 90th BMI percentile at both time points (*n* = 175, 89 girls, 86 boys) according to the German and European guidelines (Poskitt, 1995; see also Kurth and Rosario, 2007).

### Eating behavior questionnaire data

For the assessment of children's eating behavior the four “food approach” subscales emotional overeating, food responsiveness, desire to drink and enjoyment of food of the CEBQ (Wardle et al., 2001) as well as the subscale external eating of the DEBQ (Braet and Van Strien, 1997; Franzen and Florin, 1997) were used.

In case of a missing German translation, the particular item was translated into German and back translated by a native English speaker. Due to time constraints, we only included three items with the highest factor loadings according to Wardle et al. (2001) and Van Strien et al. (1986) for each scale while avoiding redundancy concerning the contents. However, the factorial structure remained the same, while the internal consistency was acceptable to good (Cronbach's α T1: 0.72–0.89; T2: 0.75–0.90).

Parents were asked to rate their child's eating behavior for the CEBQ on a five-point Likert scale (never, rarely, sometimes, often, always; 1–5) and for the DEBQ on a four-point Likert scale (never, seldom, sometimes, often; 1–4). A high score on the scales reflects higher levels of the particular eating behavior.

### Data analyses

For preliminary analyses, the Statistical Package for Social Sciences (SPSS, version 21) was used.

Then, a cross-lagged model was tested for relations between heartbeat perception and BMI and structural equation modeling (SEM) was used to evaluate the latent factor structure of the five eating styles in a measurement model as well as to further explore the relationship between heartbeat perception and eating behavior over time using maximum likelihood estimation in Mplus Version 7.10 (Muthén and Muthén, 1998-2012). By analyzing the constructs as latent variables, random measurement errors were controlled for.

To deal with missing values, we employed full-information maximum likelihood (FIML) estimation for all analyses, which has been found to be very efficient for incomplete data (Enders, 2001). In the current study, the rate of missing data was under 12.5% for children's data (HBP-Score: T1: 12.4%, T2: 11.9%; BMI: T1: 0.8%, T2: 3.1%) and under 30% for parents' data (T1: 19.4—20.3%, T2: 28.2—29.2%).

Sex was dummy coded (0 = girls, 1 = boys) and standardized coefficients for all paths were estimated.

Besides Chi-Square (χ^2^) we used Root Mean-Square Error of Approximation (RMSEA), Comparative Fit Index (CFI) and Standardized Root Mean Square Residual (SRMR) to evaluate the goodness of fit since the traditional χ^2^– Test has been shown to be highly sensitive to slight deviations from perfect fit in large samples (Brown, 2006). A RMSEA and a SRMR of 0.05 and below and a CFI of 0.90 and above indicate a good fit to the data (Hu and Bentler, 1999; Kline, 2005).

## Results

### Descriptives and correlations

Means and SDs of the main observed study variables, as well as bivariate correlations at both time points are presented in Table 1. Main results indicated that in general, BMI-SDS was positively correlated with all eating behavior styles and was negatively correlated with the educational attainment of the mother within time and across time. All of the eating behavior styles were positively inter-correlated, indicating that they all related to each other and apparently, they all represented “food approach” behavior. Age was not significantly correlated with any eating behavior, but it was slightly positively correlated with the HBP-Score at T2, so that we considered age as covariate in further latent analyses.

**Table 1:** **Descriptives and correlations among observed study variables**.

	**1**	**2**	**3**	**4**	**5**	**6**	**7**	**8**	**9**	**10**	**11**	**12**	**13**	**14**	**15**	**16**	**17**	**Mean (*SD*)**	**Range**
1. Age1	–																	8.38 (0.95)	6.25–11.33
2. Edu	−0.07^*^	–																4.81 (1.04)	1–6
3. BMI1	−0.01	−0.19^***^	–															0.15 (0.99)	−4.17–3.26
4. HBP1	−0.01	0.04	0.01	–														0.55 (0.26)	0–0.99
5. Ext1	0.01	−0.02	0.21^***^	−0.01	–													2.81 (0.67)	1–4
6. Emo1	0.02	−0.04	0.17^***^	0.03	0.24^***^	–												1.22 (0.41)	1–4
7. Resp1	0.05	−0.14^***^	0.45^***^	−0.05	0.49^***^	0.39^***^	–											1.61 (0.89)	1–5
8. Drink1	−0.01	−0.23^***^	0.19^***^	0.01	0.15^***^	0.24^***^	0.26^***^	–										1.94 (0.93)	1–5
9. Enj1	0.02	−0.01	0.25^***^	−0.07^*^	0.46^***^	0.10^***^	0.44^***^	0.05	–									3.52 (0.83)	1–5
10. Age2	0.99^***^	−0.06^*^	−0.01	−0.01	0.01	0.03	0.04	−0.01	0.01	–								9.13 (0.93)	7.12–11.91
11. BMI2	−0.05	−0.19^***^	0.91^***^	−0.01	0.20^***^	0.14^***^	0.42^***^	0.19^***^	0.24^***^	−0.04	–							0.19 (1.00)	−4.75–3.30
12. HBP2	0.05	0.05	−0.05	0.32^***^	−0.02	0.01	−0.04	−0.03	−0.03	0.05^*^	−0.04	–						0.56 (0.24)	0–1
13. Ext2	−0.01	−0.06	0.21^***^	0.02	0.58^***^	0.14^***^	0.38^***^	0.15^***^	0.35^***^	−0.01	0.20^***^	−0.03	–					2.80 (0.69)	1–4
14. Emo2	0.03	−0.10^**^	0.18^***^	−0.02	0.20^***^	0.41^***^	0.31^***^	0.15^***^	0.07^*^	0.04	0.19^***^	−0.04	0.22^***^	–				1.22 (0.39)	1–4
15. Resp2	0.01	−0.15^***^	0.47^***^	0.01	0.43^***^	0.27^***^	0.72^***^	0.23^***^	0.36^***^	0.01	0.46^***^	−0.02	0.46^***^	0.39^***^	–			1.61 (0.90)	1–5
16. Drink2	−0.02	−0.24^***^	0.18^***^	0.01	0.15^***^	0.16^***^	0.25^***^	0.67^***^	0.07^*^	−0.02	0.18^***^	−0.04	0.24^***^	0.22^***^	0.34^***^	–		1.84 (0.88)	1–5
17. Enj2	−0.01	−0.02	0.26^***^	−0.02	0.39^***^	0.07^*^	0.36^***^	0.02	0.71^***^	−0.03	0.28^***^	−0.04	0.40^***^	0.10^**^	0.43^***^	0.10^***^	–	3.56 (0.80)	1–5

1, Time 1; 2, Time 2; BMI, body mass index standard deviation score; Drink, desire to drink; Edu, educational attainment of the mother; Emo, emotional eating behavior; Enj, enjoyment of food; Ext, external eating behavior; HBP, heartbeat perception score; Resp, food responsiveness.

* p < 0.05,

** p < 0.01,

*** p < 0.001

### Group differences between overweight and normal weight children

**Figure 1:**
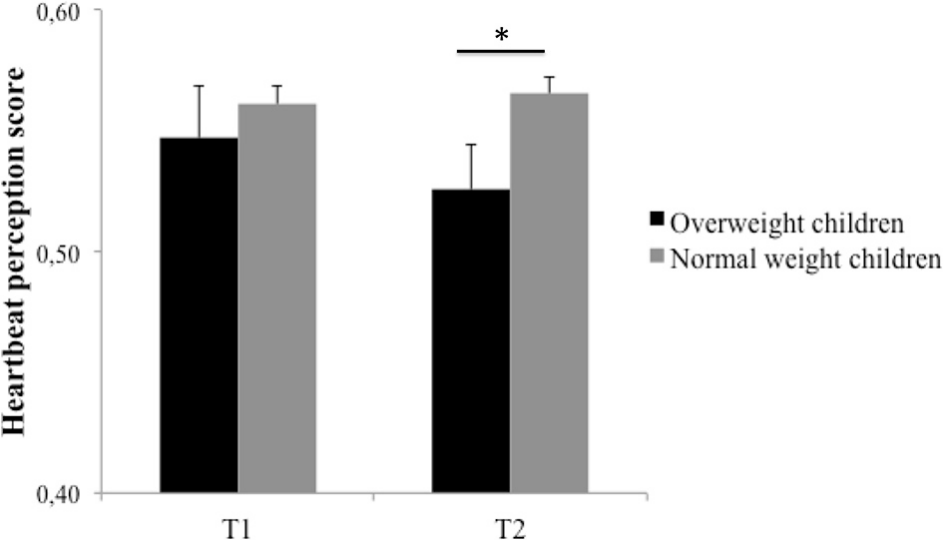
**Heartbeat perception scores for both measurement points (T1 and T2) contrasting overweight and normal weight group**. Error bars represent standard error of means. ^*^p < 0.05.

We also tested differences in the main study variables between normal weight and overweight children. As expected, results indicated that overweight children had higher mean scores on all eating behavior scales at both time points than normal weight children (*t*s ≥ 3.94, *p*s < 0.001). Further, overweight children had mothers with significantly lower educational levels than normal weight children (overweight children: *M* = 4.17, *SD* = 1.00, normal weight children: *M* = 4.91, *SD* = 0.99; *t*_(186.04)_ = 8.34, *p* < 0.001, *d* = 0.74). There were no weight status differences in age across time points and no differences in IS between both groups at T1. However, overweight children showed a significant lower HBP-Score than normal weight children at T2 (overweight children: *M* = 0.53, *SD* = 0.24, normal weight children: *M* = 0.57, *SD* = 0.24; *t*_(1259)_ = 1.98, *p*< 0.05, *d* = 0.17, see Figure 1).

### Heartbeat perception and body mass index

To examine the association of IS and BMI-SDS, we tested a cross-lagged model in which we included stability paths of BMI-SDS and HBP-Score, covariances among the residuals of the two constructs within time as well as cross-lagged paths between both, while controlling for age, sex and educational attainment of the mother. This allowed us to examine the continuity of constructs over time as well as the covariances between the constructs within and over time that are over and above what may have already occurred previous time.

**Figure 2:**
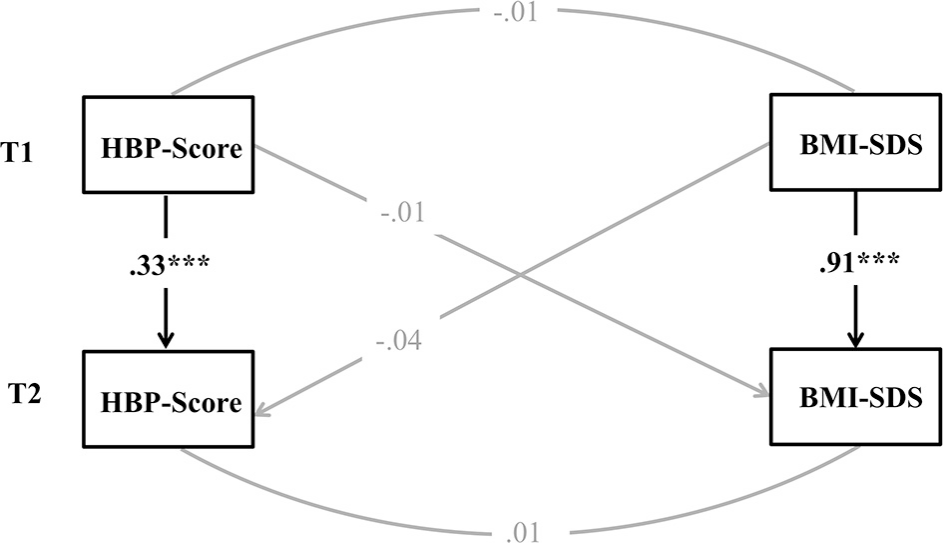
**Structural model for reciprocal time-lagged effects between heartbeat perception (HBP) and body mass index standard deviation score (BMI-SDS)**. Controlled for age, sex, and educational attainment of the mother. Displayed are standardized estimates. ^***^p < 0.001.

As can be seen in Figure 2, BMI-SDS showed a high stability over the 1-year test period (β = 0.91, *p* < 0.001), while the HBP-Score showed a relatively low stability (β = 0.33, *p* < 0.001). There was neither a cross-sectional nor a prospective association between the two variables in the sample.

### Measurement model of eating behavior

A measurement model including all eating behavior styles in one single model, in which all latent eating behaviors were allowed to covary and errors of the same indicators were allowed to covary over time, was first examined in order to determine whether the observed variables loaded onto the latent variables they are intended to measure and whether error terms related to each other in expected ways. The measurement model fit the data adequately, with χ^2^_(*df* = 345)_ = 724.82, *p* < 0.001, *RMSEA* = 0.03, *CFI* = 0.98, *SRMR* = 0.03. All manifest variables significantly loaded on their hypothesized latent variables (standardized factor loadings: 0.56–0.93, *p*s < 0.001). Next, to ensure measurement invariance of the latent constructs over time, factor loadings, intercepts and error terms were set to be equal across time. The model fit the data equally well [χ^2^_(*df* = 380)_ = 823.10, *p* < 0.001, *RMSEA* = 0.03, *CFI* = 0.98, *SRMR* = 0.03], with all fit indices remaining stable, so that we concluded from this finding of strict temporal measurement invariance, that eating behaviors were assessed equally across the study period in our sample, offering an excellent basis for longitudinal investigations involving these constructs.

### Heartbeat perception and eating behavior

In the context of SEM, we then tested reciprocal relations between HBP-Score and eating behaviors after controlling for construct stability and concurrent cross-sectional correlations. Therefore, each T2 eating behavior style was predicted by its T1 measure as well as the T1 HBP-Score, whereby the T2 HBP-Score was predicted by its T1 measure as well as all T1 eating behaviors, controlling for age, sex, educational attainment of the mother, BMI-SDS and cross-sectional relations. Model fit of this model was good [χ^2^_(*df* = 520)_ = 1211.75, *p* < 0.001, *RMSEA* = 0.03, *CFI* = 0.97, *SRMR* = 0.03], showing considerable stability of the constructs over the 1-year test period, with stability coefficients of the eating scales ranging from 0.51 to 0.77. There was a slight significant negative cross-sectional association between HBP-Score and enjoyment of food of −0.07 (*p* < 0.05) at T1. However, there was no other cross-sectional or prospective association between HBP-Score and eating behavior styles in the total sample.

**Figure 3:**
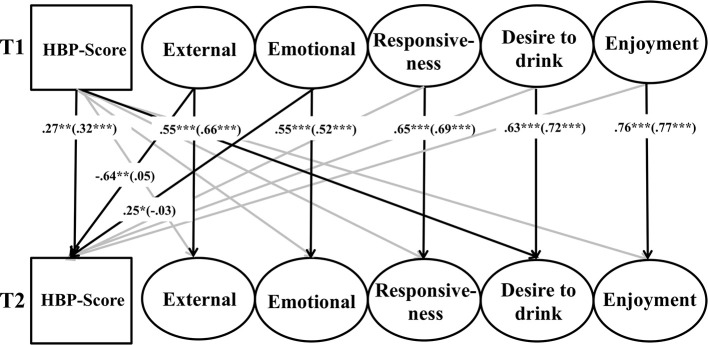
**Results of multi-group structural equation analysis evaluating the reciprocal effects between heartbeat perception and eating behavior across normal weight and overweight children**. Model fit: χ^2^_(*df* = 1040)_ = 1987.08, *p* < 0.001, *CFI* = 0.94, *RMSEA* = 0.04, *SRMR* = 0.05. Controlled for age, sex, educational attainment of the mother and cross sectional relations. For the sake of clarity, only significant standardized estimates of path coefficients over time are presented. See the text for further information on the exact measurement model. Standardized coefficients for overweight children are reported outside parentheses and standardized coefficients for normal weight children are reported inside parentheses. ^***^p < 0.001, ^**^p < 0.01, ^*^p < 0.05

Given our hypotheses involving weight status differences, we conducted multiple-group analyses comparing patterns of relations among variables in the subsamples of normal weight compared with overweight children. The multi-group approach has the advantage of being able to test the equality of parameters between groups through restrictions. The assumption of strict measurement invariance across groups was also made and again, we controlled for age, sex, educational attainment of the mother and cross-sectional relations. The result of this analysis with information on the good model fit in the notes can be found in Figure 3.

The constructs showed comparable stability values in both groups as was found in the total sample. Stabilities of the HBP-Score did not significantly differ between overweight and normal weight group (*p* = 0.52). We found no significant cross-sectional or prospective associations between eating behaviors and HBP-Score in the normal weight group. However, we found that external eating behavior at T1 was a strong negative predictor (β = −0.64, *p* < 0.01) and emotional overeating at T1 was a positive predictor (β = 0.25, *p* < 0.05) for the HBP-Score at T2 in the overweight group. By using the analysis option MODEL CONSTRAINTS, we tested the equality of both β-estimates in the groups. Results showed that both β-estimates significantly differed between normal weight and overweight children (*p* < 0.05). There was also a significant association between HBP-Score and desire to drink over time, namely that the HBP-Score at T1 was a slight positive predictor for desire to drink at T2 (β = 0.16, *p* < 0.05) in the overweight group of children. However, the direct comparison of the β-estimates between the groups did not reach significance (*p* = 0.10), so that we abstain from further interpretation of this finding.

## Discussion

The present study targeted the role of IS measured with a heartbeat perception task in relation with BMI as well as eating behavior in children over time. For the first time we showed a 1-year stability of the heartbeat perception score in children as well as prospective relations to external and emotional eating behavior in overweight children, while no association was found in normal weight children.

In agreement with earlier studies (Sleddens et al., 2008; Viana et al., 2008; Webber et al., 2009; Santos et al., 2011), we found significant stronger appetitive responses to food in overweight and obese children compared with normal weight children as indicated by higher scores on the CEBQ “food approach” scales as well as on the DEBQ external eating scale at both points of measurement. This is further support of the theory that already obese and those at risk of developing obesity exhibit heightened responses to external food cues, while ignoring internal hunger and satiety signals (Schachter, 1971; Jansen et al., 2003). Interestingly, the mother's educational level was significantly negatively related to BMI as well as to the majority of investigated eating behaviors, which once again suggests a propagated incidence of overweight in lower educated families with usually lower socio-economic states (Wang and Lim, 2012). Further, all five examined eating behaviors showed considerable stability over the 1-year testing interval and no associations with age, indicating an already reasonable stable eating pattern in elementary school children.

We found no direct relation between IS and BMI, neither cross-sectionally nor prospectively. This is in line with most other studies (e.g., Gardner et al., 1990; Khalsa et al., 2009; Koch and Pollatos, 2014), except of Herbert et al. (2013) who found a negative association. However, the authors included only adult female participants with a relatively limited BMI range of 20–25. Nevertheless, by directly comparing normal and overweight children in the current study, overweight children showed significantly lower IS only at T2, which might indicate a later developing influence of higher body weight on interoceptive processes or maybe a non-linear relation we were not able to detect in the total model and which might deserve further investigation over time. Since lower IS could be demonstrated in adult overweight (Herbert and Pollatos, 2014), changes in IS in the first elementary school years might reflect influential factors for stability and increase of overweight into adulthood.

Relating to our second main question of the study, we could show that in a model that considers various “food approach” behaviors, no meaningful cross-sectional or prospective associations between IS and eating behaviors were found in the total sample or in normal weight children. However, when applying the same model in overweight children compared to normal weight children, external eating turned out to be a negative predictor and emotional eating turned out to be a positive predictor for later IS in overweight children only. Since we found those prospective relations only in overweight children, who also showed the general tendency to score higher on “food approach” behaviors, it appears that exceeding a critical “threshold” in rather problematic eating seems to be necessary in order to diminish the confidence in detecting bodily sensations over time. More precisely, one might interpret the prospective negative relation of external eating and heartbeat perception in line with externality theory (Schachter, 1968, 1971; Rodin, 1981) and thus as a developing perceptual deficit of internal signals or a decreased internal locus of control when eating primarily externally driven.

In contrast, it seems rather surprising that emotional overeating was a prospective positive predictor for later IS. But referring to the psychosomatic theory (Bruch, 1964), this association could be seen as a developing “interpretation” deficit, as according to this theory, emotional eating primarily takes place as escape from aversive physical sensations which are “misinterpreted” as hunger or that is to say (over)eating is used as an appearing useful emotion regulation strategy in the short term. Previous research could repeatedly show that emotionally cued eaters can be characterized as having more emotional and psychological disturbances and negative self-feelings of physical competence (e.g., Van Strien et al., 1995; Braet et al., 1997; Macht, 2008). Equally, studies found that particular children with obesity report decreased levels of self-esteem, higher levels of sadness, loneliness and nervousness compared to normal weight children (Strauss, 2000; Wardle and Cooke, 2005; Puhl and Latner, 2007), which might be especially crucial for the critical age of school entrance when obesity prevalence rates significantly increase (Yoshinaga et al., 2004; Nader et al., 2006).

Therefore, research shows that overweight children frequently experience negative emotional states, which might give them the “permission” to eat and as a consequence, a higher alertness for those frequently occurring negative conditions of the body seems to take place over time. Another similar possible explanation for the relation between emotional eating and higher scores on the HBP-task in overweight children might be the fact that earlier studies found that both IS (Eley et al., 2004, 2007; Domschke et al., 2010) as well as emotional eating (Van Strien et al., 1995; Goossens et al., 2009) are related to greater anxiety in adults and children. So it might be that higher levels of emotional eating lead to higher anxiety as the children rely on this type of coping method that provides short term relief but no effective help and maybe increased anxiety symptoms in the long term. So, the possible moderating or mediating role of anxiety might be worth to consider in future studies.

Hence, according to our results and to what Bruch (1973) postulated, it might be possible that altered sensitivity for bodily signals and thus a decreased confidence in as well as deficits in handling those signals result from already existing rather food approach eating patterns that are a consequence of faulty learning experiences early in infancy, when caretakers do not provide food merely to assuage hunger, but in response to all expressions of e.g., distress or negative emotionality (Snoek et al., 2007; Van Strien and Bazelier, 2007; Kröller et al., 2013). For the first time, the present study suggests to give an answer about the possible direction of these relations, namely that altered perception of body signals actually rather follows non-adaptive eating behavior patterns in overweight children.

However, it is important to note that when considering the positive association between emotional eating and heartbeat perception, it appears not to be enough to perceive interoceptive signals adequately, but the results suggest that the further handling of these signals (e.g., drawing attention to them or appraising them as negative or positive and act accordingly in order to manage them) seems to be a separate essential process that seems to be determined by (over)eating behavior. This is in line with results of Herbert et al. (2013) showing that IS and the subjective appraisal of interoceptive signals as aversive or pleasant were independently associated with intuitive eating behavior. The authors also explained their findings by stating that individual interoception accuracy and the evaluation of these body signals are independent processes, both related to eating according to one's needs and not because of emotional states or external distractors. Hence, it seems reasonable that Pollatos et al. (2008) and Eshkevari et al. (2014) did not find an association between IS (measured via heartbeat perception) and IA (measured via the corresponding EDI subscale), since even according to our results, sensitivity toward internal bodily signals does not necessarily imply a correct identification or interpretation of e.g., emotions or hunger and therefore a “correct” acting according to these signals or vice versa (Mehling et al., 2009). But without further follow-up studies on the sample used in this study this assumption remains of speculative nature. Future investigations could also benefit from including data on both, IS and IA, in order to further explore this supposition and thus the question of interoceptive deficits being a perceptual or rather a cognitive relevant factor for eating and weight problems.

A good broader candidate for explaining the found divergent associations of IS with external and emotional eating only in overweight is the escape theory of eating by Heatherton and Baumeister (1991). It incorporates elements of externality and psychosomatic theory. Although originally postulated for binge eaters, this theory might also apply for more general non-clinical overeating. The authors proposed that eating is motivated by a desire to escape from self-awareness which may be characterized by low self-esteem together with a high self-focus resulting from difficult (perceived) expectations and standards. Low levels of thinking and emotional distress (anxiety and depression) occur, which according to this theory result in a dampening of affect and at the same time in an attention narrowing and reliance on immediate environmental stimuli. These assumptions have been supported by research suggesting that a state of uncontrollable negative affects enhances the reactions of overweight persons to external cues (Slochower et al., 1981; Slochower, 1983). So, in this way, emotions and environment may operate conjointly to produce overeating, which relating to our results, is expressed in a higher self-focus when eating emotionally induced and a lower self-focus when eating externally driven. Interestingly, none of the other “food approach” behaviors was found to be directly related to IS, indicating that these eating styles are important characteristics for the description of (over)eating patterns in overweight children, but do not seem to directly affect IS.

Our study should be placed within the context of strengths and limitations of the used design. First, we examined a large representative sample with respect to sex and weight status distribution of children. To our knowledge, it is the first study to longitudinally evaluate the relationship between IS, BMI and eating behavior in children in a well-controlled and low measurement error implying analysis. However, we used subjective parent ratings of eating behavior through questionnaires, which might be considered as rather uncertain or biased, while the direct measurement of eating behavior in children could be even more illuminating in further studies (Borrell, 2011). Besides that, although usually showing reliable results (Wardle et al., 2001) parent report of eating behavior might differ from self-report of children, which could also interrelate with the variables of this study. It could further be interesting to investigate sex-based differences in the relation between IS and “food approach behavior” in overweight, since especially girls and females were found to be prone to eat when perceiving stress, worries and tension (Torres and Nowson, 2007; Nguyen-Rodriguez et al., 2009). Unfortunately, the sample size in this study was too small to reliably explore this question on a latent basis with the set up model. Since heartbeat perception showed only a relatively low stability in the child sample and no longitudinal comparison to other studies is feasible, we can only speculate about possible underlying developmental changes during puberty. It would be interesting to further observe this development and its relation to eating and weight disorder trends in the time interval of adolescence when vulnerability to these problems increases.

We conclude that, while no direct longitudinal association of IS with body mass index seems to exist in children, only in overweight, external and emotional eating behavior can be predictive for later IS, whereas no such relation can be found in normal weight children. As intervention programs for overweight children and adults that focus on the appreciation of and confidence in one's body signals as well as appetite awareness and mindfulness (e.g., Bacon et al., 2005; Daubenmier et al., 2011; Bloom et al., 2013) have shown promise for the treatment of overweight and obesity, this study gives further support of the relevance of this topic, although showing that altered IS follows dysfunctional behavior. So, more than this, our results underline that overweight and obese children should learn other coping mechanisms than eating when faced with emotional arousal and/or external food cues in order not to fall in a higher alertness toward negative emotional/bodily states or in an complete attentional withdrawal from own body signals and as an result in an orientation toward external signals.

## Author contributions

Anne Koch and Olga Pollatos both designed the study and supervised all actions taken within this study. Anne Koch managed the literature searches, data collection as well as data analysis and drafted the manuscript. Both authors contributed to and have approved the final manuscript.

### Conflict of interest statement

The authors declare that the research was conducted in the absence of any commercial or financial relationships that could be construed as a potential conflict of interest.

## References

[B1] Ainley V. Tajadura-Jiménez A. Fotopoulou A. Tsakiris M. (2012). Looking into myself: changes in interoceptive sensitivity during mirror self-observation. Psychophysiology 49, 1504–1508 10.1111/j.1469-8986.2012.01468.x 22978299 PMC3755258

[B2] Ainley V. Tsakiris M. (2013). Body conscious? Interoceptive awareness, measured by heartbeat perception, is negatively correlated with self-objectification. PLoS ONE 8:e55568 10.1371/journal.pone.0055568 23405173 PMC3565964

[B3] Bacon L. Stern J. S. Van Loan M. D. Keim N. L. (2005). Size acceptance and intuitive eating improve health for obese, female chronic dieters. J. Am. Diet. Assoc. 105, 929–936 10.1016/j.jada.2005.03.011 15942543

[B4] Bechara A. Naqvi N. (2004). Listening to your heart: interoceptive awareness as a gateway to feeling. Nat. Neurosci. 7, 102–103 10.1038/nn0204-102 14747831

[B5] Bizeul C. Sadowsky N. Rigaud D. (2001). The prognostic value of initial EDI scores in anorexia nervosa patients: a prospective follow-up study of 5–10 years. Eur. Psychiatry 16, 232–238 10.1016/S0924-9338(01)00570-3 11418274

[B6] Bloom T. Sharpe L. Mullan B. Zucker N. (2013). A pilot evaluation of appetite-awareness training in the treatment of childhood overweight and obesity: a preliminary investigation. Int. J. Eat. Disord. 46, 47–51 10.1002/eat.22041 22826019

[B7] Borrell B. (2011). Epidemiology: every bite you take. Nat. News 470, 320–322 10.1038/470320a 21331018

[B8] Braet C. Mervielde I. Vandereycken W. (1997). Psychological aspects of childhood obesity: a controlled study in a clinical and nonclinical sample. J. Pediatr. Psychol. 22, 59–71 10.1093/jpepsy/22.1.59 9019048

[B9] Braet C. Van Strien T. (1997). Assessment of emotional, externally induced and restrained eating behaviour in nine to twelve-year-old obese and non-obese children. Behav. Res. Ther. 35, 863–873 10.1016/S0005-7967(97)00045-4 9299807

[B10] Brown T. A. (2006). Confirmatory Factor Analysis for Applied Research. New York, NY: The Guilford Press

[B11] Bruch H. (1964). Psychological aspects of overeating and obesity. Psychosomatics 5, 269–274 14235740 10.1016/s0033-3182(64)72385-7

[B12] Bruch H. (1973). Eating Disorders: Obesity, Anorexia Nervosa, and the Person Within. New York, NY: Basic Books

[B13] Caccialanza R. Nicholls D. Cena H. Maccarini L. Rezzani C. Antonioli L. (2004). Validation of the Dutch Eating Behaviour Questionnaire parent version (DEBQ-P) in the Italian population: a screening tool to detect differences in eating behaviour among obese, overweight and normal-weight preadolescents. Eur. J. Clin. Nutr. 58, 1217–1222 10.1038/sj.ejcn.1601949 15054434

[B14] Clausen L. Rosenvinge J. H. Friborg O. Rokkedal K. (2011). Validating the Eating Disorder Inventory-3 (EDI-3): a comparison between 561 female eating disorders patients and 878 females from the general population. J. Psychopathol. Behav. Assess. 33, 101–110 10.1007/s10862-010-9207-4 21472023 PMC3044826

[B15] Craig A. D. (2002). How do you feel? Interoception: the sense of the physiological condition of the body. Nat. Rev. Neurosci. 3, 655–666 10.1038/nrn894 12154366

[B16] Craig A. D. (2009). How do you feel—now? The anterior insula and human awareness. Nat. Rev. Neurosci. 10, 59–70 10.1038/nrn2555 19096369

[B17] Daubenmier J. Kristeller J. Hecht F. M. Maninger N. Kuwata M. Jhaveri K. (2011). Mindfulness intervention for stress eating to reduce cortisol and abdominal fat among overweight and obese women: an exploratory randomized controlled study. J. Obes. 2011:e651936 10.1155/2011/651936 21977314 PMC3184496

[B18] Domschke K. Stevens S. Pfleiderer B. Gerlach A. L. (2010). Interoceptive sensitivity in anxiety and anxiety disorders: an overview and integration of neurobiological findings. Clin. Psychol. Rev. 30, 1–11 10.1016/j.cpr.2009.08.008 19751958

[B19] Dunn B. D. Dalgleish T. Ogilvie A. D. Lawrence A. D. (2007). Heartbeat perception in depression. Behav. Res. Ther. 45, 1921–1930 10.1016/j.brat.2006.09.008 17087914

[B20] Eberenz K. P. Gleaves D. H. (1994). An examination of the internal consistency and factor structure of the eating disorder inventory-2 in a clinical sample. Int. J. Eat. Disord. 16, 371–379 7866416 10.1002/1098-108x(199412)16:4<371::aid-eat2260160406>3.0.co;2-w

[B21] Eley T. C. Gregory A. M. Clark D. M. Ehlers A. (2007). Feeling anxious: a twin study of panic/somatic ratings, anxiety sensitivity and heartbeat perception in children. J. Child Psychol. Psychiatry 48, 1184–1191 10.1111/j.1469-7610.2007.01838.x 18093023

[B22] Eley T. C. Stirling L. Ehlers A. Gregory A. M. Clark D. M. (2004). Heart-beat perception, panic/somatic symptoms and anxiety sensitivity in children. Behav. Res. Ther. 42, 439–448 10.1016/S0005-7967(03)00152-9 14998737

[B23] Enders C. K. (2001). The performance of the full information maximum likelihood estimator in multiple regression models with missing data. Educ. Psychol. Meas. 61, 713–740 10.1177/0013164401615001

[B24] Eshkevari E. Rieger E. Musiat P. Treasure J. (2014). An investigation of interoceptive sensitivity in eating disorders using a heartbeat detection task and a self-report measure. Eur. Eat. Disord. Rev. 22, 383–388 10.1002/erv.2305 24985151

[B25] Fassino S. Pierò A. Gramaglia C. Abbate-Daga G. (2004). Clinical, psychopathological and personality correlates of interoceptive awareness in anorexia nervosa, bulimia nervosa and obesity. Psychopathology 37, 168–174 10.1159/000079420 15237246

[B26] Franzen S. Florin I. (1997). Der Dutch Eating Behavior Questionnaire für Kinder (DEBQ-K) – Ein Fragebogen zur Erfassung gezügelten Essverhaltens. Kindheit Entwicklung 6, 116–122

[B27] Füstös J. Gramann K. Herbert B. M. Pollatos O. (2013). On the embodiment of emotion regulation: interoceptive awareness facilitates reappraisal. Soc. Cogn. Affect. Neurosci. 8, 911–917 10.1093/scan/nss089 22933520 PMC3831556

[B28] Gamelin F.-X. Baquet G. Berthoin S. Bosquet L. (2008). Validity of the Polar S810 to measure R-R intervals in children. Int. J. Sports Med. 29, 134–138 10.1055/s-2007-964995 17614016

[B29] Gardner R. M. Morrell J. A. Jr. Watson D. N. Sandoval S. L. (1990). Cardiac self-perception in obese and normal persons. Percept. Mot. Skills 70, 1179–1186 2399094

[B30] Garfinkel S. N. Critchley H. D. (2013). Interoception, emotion and brain: new insights link internal physiology to social behaviour. Commentary on: “Anterior insular cortex mediates bodily sensibility and social anxiety” by Terasawa et al. (2012). Soc Cogn. Affect Neurosci. 8, 231–234 10.1093/scan/nss140 23482658 PMC3594730

[B31] Garner D. M. (1991). The Eating Disorder Inventory-C. Lutz, FL, ON: Psychological Assessment Resources, Inc.

[B32] Garner D. M. (2004). The Eating Disorder Inventory-3 (EDI-3). *Professional Manual* Odessa, FL, ON: Psychological Assessment Resources

[B33] Garner D. M. Olmstead M. P. Polivy J. (1983). Development and validation of a multidimensional eating disorder inventory for anorexia nervosa and bulimia. Int. J. Eat. Disord. 2, 15–34 10.1002/1098-108X(198321)2:2<15::AID-EAT2260020203>3.0.CO;2-617147952

[B34] Golay A. Hagon I. Painot D. Rouget P. Allaz A. F. Morel Y. (1997). Personalities and alimentary behaviors in obese patients. Patient Educ. Couns. 31, 103–112 10.1016/S0738-3991(97)00995-6 9216351

[B35] Goossens L. Braet C. Van Vlierberghe L. Mels S. (2009). Loss of control over eating in overweight youngsters: the role of anxiety, depression and emotional eating. Eur. Eat. Disord. Rev. 17, 68–78 10.1002/erv.892 18729132

[B36] Gustafsson S. A. Edlund B. Kjellin L. Norring C. (2010). Characteristics measured by the eating disorder inventory for children at risk and protective factors for disordered eating in adolescent girls. Int. J. Women's Health 2, 375–379 10.2147/IJWH.S12349 21151684 PMC2990906

[B37] Heatherton T. F. Baumeister R. F. (1991). Binge eating as escape from self-awareness. Psychol. Bull. 110, 86–108 10.1037/0033-2909.110.1.86 1891520

[B38] Herbert B. M. Blechert J. Hautzinger M. Matthias E. Herbert C. (2013). Intuitive eating is associated with interoceptive sensitivity. Effects on body mass index. Appetite 70, 22–30 10.1016/j.appet.2013.06.082 23811348

[B39] Herbert B. M. Herbert C. Pollatos O. Weimer K. Enck P. Sauer H. (2012a). Effects of short-term food deprivation on interoceptive awareness, feelings and autonomic cardiac activity. Biol. Psychol. 89, 71–79 10.1016/j.biopsycho.2011.09.004 21958594

[B40] Herbert B. M. Muth E. R. Pollatos O. Herbert C. (2012b). Interoception across modalities: on the relationship between cardiac awareness and the sensitivity for gastric functions. PLoS ONE 7:e36646 10.1371/journal.pone.0036646 22606278 PMC3350494

[B41] Herbert B. M. Pollatos O. (2012). The body in the mind: on the relationship between interoception and embodiment. Top. Cogn. Sci. 4, 692–704 10.1111/j.1756-8765.2012.01189.x 22389201

[B42] Herbert B. M. Pollatos O. (2014). Attenuated interoceptive sensitivity in overweight and obese individuals. Eat. Behav. 3, 445–448 10.1016/j.eatbeh.2014.06.002 25064297

[B43] Herbert B. M. Ulbrich P. Schandry R. (2007). Interoceptive senstivity and physical effort: implications for the self-control of physical load in everyday life. Psychophysiology 44, 194–202 10.1111/j.1469-8986.2007.00493.x 17343703

[B44] Hu L. Bentler P. M. (1999). Cutoff criteria for fit indexes in covariance structure analysis: conventional criteria versus new alternatives. Struct. Equ. Model. A Multidisciplinary J. 6, 1–55 10.1080/10705519909540118

[B45] Jacobi C. Hayward C. de Zwaan M. Kraemer H. C. Stewart W. (2004). Coming to terms with risk factors for eating disorders: application of risk terminology and suggestions for a general taxonomy. Psychol. Bull. 130, 19–65 10.1037/0033-2909.130.1.19 14717649

[B46] Jansen A. Theunissen N. Slechten K. Nederkoorn C. Boon B. Mulkens S. (2003). Overweight children overeat after exposure to food cues. Eat. Behav. 4, 197–209 10.1016/S1471-0153(03)00011-4 15000982

[B47] Khalsa S. S. Rudrauf D. Tranel D. (2009). Interoceptive awareness declines with age. Psychophysiology 46, 1130–1136 10.1111/j.1469-8986.2009.00859.x 19602175 PMC2865139

[B48] Killen J. D. Taylor C. B. Hayward C. Haydel K. F. Wilson D. M. Hammer L. (1996). Weight concerns influence the development of eating disorders: a 4-year prospective study. J. Consult. Clin. Psychol. 64, 936–940 10.1037/0022-006X.64.5.936 8916622

[B49] Killen J. D. Taylor C. B. Hayward C. Wilson D. M. Haydel K. F. Hammer L. D. (1994). Pursuit of thinness and onset of eating disorder symptoms in a community sample of adolescent girls: a three-year prospective analysis. Int. J. Eat. Disord. 16, 227–238 7833956 10.1002/1098-108x(199411)16:3<227::aid-eat2260160303>3.0.co;2-l

[B50] Kingsley M. Lewis M. J. Marson R. E. (2005). Comparison of Polar 810s and an ambulatory ECG system for RR interval measurement during progressive exercise. Int. J. Sports Med. 26, 39–44 10.1055/s-2004-817878 15643533

[B51] Klabunde M. Acheson D. T. Boutelle K. N. Matthews S. C. Kaye W. H. (2013). Interoceptive sensitivity deficits in women recovered from bulimia nervosa. Eat. Behav. 14, 488–492 10.1016/j.eatbeh.2013.08.002 24183142 PMC3817494

[B52] Kline R. B. (2005). Principles and Practice of Structural Equation Modeling. New York, NY: The Guilford

[B53] Koch A. Pollatos O. (2014). Cardiac sensitivity in children: sex differences and its relationship to parameters of emotional processing. Psychophysiology 51, 932–941 10.1111/psyp.12233 24810627

[B54] Kröller K. Jahnke D. Warschburger P. (2013). Are maternal weight, eating and feeding practices associated with emotional eating in childhood? Appetite 65, 25–30 10.1016/j.appet.2012.11.032 23380038

[B55] Kromeyer-Hauschild K. Wabitsch M. Kunze D. Geller F. Geiß H. C. Hesse V. (2001). Perzentile für den body-mass-index für das Kindes- und Jugendalter unter Heranziehung verschiedener deutscher Stichproben.[Percentiles of body mass index in children and adolescents evaluated from different regional German studies.] Monatsschrift Kinderheilkunde 149, 807–818 10.1007/s001120170107

[B56] Kurth D. B.-M. Rosario A. S. (2007). Die Verbreitung von Übergewicht und Adipositas bei kindern und Jugendlichen in deutschland [The prevalence of overweight and obese children and adolescents living in Germany. Results of the German Health Interview and Examination Survey for Children and Adolescents (KiGGS)]. Ernährung Wissenschaft und Praxis 1, 213–219 10.1007/s12082-007-0050-2 17514458

[B57] Leon G. R. Fulkerson J. A. Perry C. L. Cudeck R. (1993). Personality and behavioral vulnerabilities associated with risk status for eating disorders in adolescent girls. J. Abnorm. Psychol. 102, 438–444 10.1037/0021-843X.102.3.438 8408956

[B58] Leon G. R. Fulkerson J. A. Perry C. L. Early-Zald M. B. (1995). Prospective analysis of personality and behavioral vulnerabilities and gender influences in the later development of disordered eating. J. Abnorm. Psychol. 104, 140–149 10.1037/0021-843X.104.1.140 7897036

[B59] Leon G. R. Fulkerson J. A. Perry C. L. Keel P. K. Klump K. L. (1999). Three to four year prospective evaluation of personality and behavioral risk factors for later disordered eating in adolescent girls and boys. J. Youth Adolesc. 28, 181–196 10.1023/A:1021649314458

[B60] Macht M. (2008). How emotions affect eating: a five-way model. Appetite 50, 1–11 10.1016/j.appet.2007.07.002 17707947

[B61] Matsumoto R. Kitabayashi Y. Narumoto J. Wada Y. Okamoto A. Ushijima Y. (2006). Regional cerebral blood flow changes associated with interoceptive awareness in the recovery process of anorexia nervosa. Prog. Neuropsychopharmacol. Biol. Psychiatry 30, 1265–1270 10.1016/j.pnpbp.2006.03.042 16777310

[B62] Mehling W. E. Gopisetty V. Daubenmier J. Price C. J. Hecht F. M. Stewart A. (2009). Body awareness: construct and self-report measures. PLoS ONE 4:e5614 10.1371/journal.pone.0005614 19440300 PMC2680990

[B63] Merwin R. M. Zucker N. L. Lacy J. L. Elliott C. A. (2010). Interoceptive awareness in eating disorders: distinguishing lack of clarity from non-acceptance of internal experience. Cogn. Emot. 24, 892–902 10.1080/02699930902985845

[B64] Muthén L. K. Muthén B. O. (1998 - 2012). Mplus User's Guide. 7th Edn. Los Angeles, CA: Muthén & Muthén

[B65] Nader P. R. O'Brien M. Houts R. Bradley R. Belsky J. Crosnoe R. (2006). Identifying risk for obesity in early childhood. Pediatrics 118, e594–e601 10.1542/peds.2005-2801 16950951

[B66] Nguyen-Rodriguez S. T. Unger J. B. Spruijt-Metz D. (2009). Psychological determinants of emotional eating in adolescence. Eat. Disord. 17, 211–224 10.1080/10640260902848543 19391020 PMC2859040

[B67] Niskanen J.-P. Tarvainen M. P. Ranta-aho P. O. Karjalainen P. A. (2004). Software for advanced HRV analysis. Comput. Methods Prog. Biomed. 76, 73–81 10.1016/j.cmpb.2004.03.004 15313543

[B68] Nunan D. Jakovljevic D. G. Donovan G. Hodges L. D. Sandercock G. R. H. Brodie D. A. (2008). Levels of agreement for RR intervals and short-term heart rate variability obtained from the Polar S810 and an alternative system. Eur. J. Appl. Physiol. 103, 529–537 10.1007/s00421-008-0742-6 18427831

[B69] Pollatos O. Kirsch W. Schandry R. (2005). On the relationship between interoceptive awareness, emotional experience and brain processes. Cogn. Brain Res. 25, 948–962 10.1016/j.cogbrainres.2005.09.019 16298111

[B70] Pollatos O. Kurz A.-L. Albrecht J. Schreder T. Kleemann A. M. Schöpf V. (2008). Reduced perception of bodily signals in anorexia nervosa. Eat. Behav. 9, 381–388 10.1016/j.eatbeh.2008.02.001 18928900

[B71] Pollatos O. Traut-Mattausch E. Schandry R. (2009). Differential effects of anxiety and depression on interoceptive accuracy. Depress. Anxiety 26, 167–173 10.1002/da.20504 19152366

[B72] Poskitt E. (1995). Defining childhood obesity: the relative body mass index (BMI). Acta Pediatr. 84, 961–963 7488831 10.1111/j.1651-2227.1995.tb13806.x

[B73] Puhl R. M. Latner J. D. (2007). Stigma, obesity, and the health of the nation's children. Psychol. Bull. 133, 557–580 10.1037/0033-2909.133.4.557 17592956

[B74] Radespiel-Tröger M. Rauh R. Mahlke C. Gottschalk T. Mück-Weymann M. (2003). Agreement of two different methods for measurement of heart rate variability. Clin. Auton. Res. 13, 99–102 10.1007/s10286-003-0085-7 12720094

[B75] Rodin J. (1981). Current status of the internal–external hypothesis for obesity: what went wrong? Am. Psychol. 36, 361–372 10.1037/0003-066X.36.4.361 7023303

[B76] Santos J. L. Ho-Urriola J. A. González A. Smalley S. V. Domínguez-Vásquez P. Cataldo R. (2011). Association between eating behavior scores and obesity in Chilean children. Nutr. J. 10, 108 10.1186/1475-2891-10-108 21985269 PMC3213088

[B77] Schachter S. (1968). Obesity and eating. Science 161, 751–756 10.1126/science.161.3843.751 5663800

[B78] Schachter S. (1971). Some extraordinary facts about obese humans and rats. Am. Psychol. 26, 129–144 10.1037/h0030817 5541215

[B79] Schandry R. (1981). Heart beat perception and emotional experience. Psychophysiology 18, 483–488 10.1111/j.1469-8986.1981.tb02486.x 7267933

[B80] Sim L. Zeman J. (2004). Emotion awareness and identification skills in adolescent girls with bulimia nervosa. J. Clin. Child Adolesc. Psychol. 33, 760–771 10.1207/s15374424jccp3304_11 15498743

[B81] Sleddens E. F. Kremers S. P. Thijs C. (2008). The children's eating behaviour questionnaire: factorial validity and association with body mass index in Dutch children aged 6–7. Int. J. Behav. Nutr. Phys. Act. 5, 49 10.1186/1479-5868-5-49 18937832 PMC2612017

[B82] Slochower J. A. (1983). Excessive Eating: the Role of Emotions and Environment. New York, NY: Human Sciences Press

[B83] Slochower J. Kaplan S. P. Mann L. (1981). The effects of life stress and weight on mood and eating. Appetite 2, 115–125 10.1016/S0195-6663(81)80005-0 7337441

[B84] Snoek H. M. Engels R. C. M. E. Janssens J. M. A. M. van Strien T. (2007). Parental behaviour and adolescents' emotional eating. Appetite 49, 223–230 10.1016/j.appet.2007.02.004 17391806

[B85] Strauss R. S. (2000). Childhood obesity and self-esteem. Pediatrics 105, e15 10.1542/peds.105.1.e15 10617752

[B86] Terasawa Y. Fukushima H. Umeda S. (2013). How does interoceptive awareness interact with the subjective experience of emotion? An fMRI Study. Hum. Brain Mapp. 34, 598–612 10.1002/hbm.21458 22102377 PMC6870042

[B87] Torres S. J. Nowson C. A. (2007). Relationship between stress, eating behavior, and obesity. Nutrition 23, 887–894 10.1016/j.nut.2007.08.008 17869482

[B88] Vaitl D. (1996). Interoception. Biol. Psychol. 42, 1–27 10.1016/0301-0511(95)05144-9 8770368

[B89] Van Strien T. (2000). Ice-cream consumption, tendency toward overeating, and personality. Int. J. Eat. Disord. 28, 460–464 10.1002/1098-108X(200012)28:4<460::AID-EAT16>3.0.CO;2-A 11054795

[B90] Van Strien T. Bazelier F. G. (2007). Perceived parental control of food intake is related to external, restrained and emotional eating in 7–12-year-old boys and girls. Appetite 49, 618–625 10.1016/j.appet.2007.03.227 17512089

[B91] Van Strien T. Frijters J. E. R. Bergers G. P. A. Defares P. B. (1986). The Dutch Eating Behavior Questionnaire (DEBQ) for assessment of restrained, emotional, and external eating behavior. Int. J. Eat. Disord. 5, 295–315 10.1002/1098-108X(198602)5:2<295::AID-EAT2260050209>3.0.CO;2-T.

[B92] Van Strien T. Herman C. P. Verheijden M. W. (2009). Eating style, overeating, and overweight in a representative Dutch sample. Does external eating play a role? Appetite 52, 380–387 10.1016/j.appet.2008.11.010 19100301

[B93] Van Strien T. Oosterveld P. (2008). The children's DEBQ for assessment of restrained, emotional, and external eating in 7- to 12-year-old children. Int. J. Eat. Disord. 41, 72–81 10.1002/eat.20424 17634965

[B94] Van Strien T. Schippers G. M. Cox W. M. (1995). On the relationship between emotional and external eating behavior. Addict. Behav. 20, 585–594 10.1016/0306-4603(95)00018-8 8712056

[B95] Viana V. Sinde S. Saxton J. C. (2008). Children's eating behaviour questionnaire: associations with BMI in Portuguese children. Br. J. Nutr. 100, 445–450 10.1017/S0007114508894391 18275626

[B96] Wang Y. Lim H. (2012). The global childhood obesity epidemic and the association between socio-economic status and childhood obesity. Int. Rev. Psychiatry 24, 176–188 10.3109/09540261.2012.688195 22724639 PMC4561623

[B97] Wardle J. Cooke L. (2005). The impact of obesity on psychological well-being. Best Pract. Res. Clin. Endocrinol. Metab. 19, 421–440 10.1016/j.beem.2005.04.006 16150384

[B98] Wardle J. Guthrie C. A. Sanderson S. Rapoport L. (2001). Development of the children's eating behaviour questionnaire. J. Child Psychol. Psychiatry. 42, 963–970 10.1111/1469-7610.00792 11693591

[B99] Webber L. Hill C. Saxton J. Van Jaarsveld C. Wardle J. (2009). Eating behaviour and weight in children. Int. J. Obes. 33, 21–28 10.1038/ijo.2008.219 19002146 PMC2817450

[B100] Whitehead W. E. Drescher V. M. (1980). Perception of gastric contractions and self-control of gastric motility. Psychophysiology 17, 552–558 10.1111/j.1469-8986.1980.tb02296.x 7443922

[B101] Wiens S. (2005). Interoception in emotional experience. Curr. Opin. Neurol. 18, 442–447 10.1097/01.wco.0000168079.92106.99 16003122

[B102] Windmann S. Schonecke O. W. Fröhlig G. Maldener G. (1999). Dissociating beliefs about heart rates and actual heart rates in patients with cardiac pacemakers. Psychophysiology 36, 339–342 10352557 10.1017/s0048577299980381

[B103] Yoshinaga M. Shimago A. Koriyama C. Nomura Y. Miyata K. Hashiguchi J. (2004). Rapid increase in the prevalence of obesity in elementary school children. Int. J. Obes. Relat. Metab. Disord. 28, 494–499 10.1038/sj.ijo.0802608 14993912

